# Parallel boosting neural network with mutual information for day-ahead solar irradiance forecasting

**DOI:** 10.1038/s41598-025-95891-1

**Published:** 2025-04-04

**Authors:** Ubaid Ahmed, Anzar Mahmood, Ahsan Raza Khan, Levin Kuhlmann, Khurram Saleem Alimgeer, Sohail Razzaq, Imran Aziz, Amin Hammad

**Affiliations:** 1 Department of Electrical Engineering, Mirpur University of Science and Technology (MUST), Mirpur, 10250 Pakistan; 2 James Watt School of Engineering, University of Glasgow, Glasgow, G128QQ UK; 3 Department of Data Science and AI, Faculty of Information Technology, Monash University, Room 273, Woodside Building, Clayton Campus, Clayton, Australia; 4 Department of Electrical and Computer Engineering, COMSATS University Islamabad, Islamabad, 45550 Pakistan; 5 Faculty of Information and Technology, Majan University College, Muscat, Sultanate of Oman; 6 Department of Physics and Astronomy, Uppsala University, P.O Box: 75120, Uppsala, Sweden; 7 Concordia Institute for Information Systems Engineering, Concordia University, Montreal, QC Canada

**Keywords:** Neural networks, Integrated approach, Parallel computing, Dimensionality reduction, Solar irradiance forecasting, Information technology, Energy science and technology, Engineering

## Abstract

The transition to sustainable energy has become imperative due to the depletion of fossil fuels. Solar energy presents a viable alternative owing to its abundance and environmental benefits. However, the intermittent nature of solar energy requires accurate forecasting of solar irradiance (SI) for reliable operation of photovoltaics (PVs) integrated systems. Traditional deep learning (DL) models and decision tree (DT)-based algorithms have been widely employed for this purpose. However, DL models often demand substantial computational resources and large datasets, while DT algorithms lack generalizability. To address these limitations, this study proposes a novel parallel boosting neural network (PBNN) framework that integrates boosting algorithms with a feedforward neural network (FFNN). The proposed framework leverages three boosting DT algorithms, Extreme Gradient Boosting (XgBoost), Categorical Boosting (CatBoost), and Random Forest (RF) regressors as base learners, operating in parallel. The intermediary forecasts from these base learners are concatenated and input into the FFNN, which assigns optimal weights to generate the final prediction. The proposed PBNN is trained and evaluated on two geographical datasets and compared with state-of-the-art techniques. The mutual information (MI) algorithm is implemented as a feature selection technique to identify the most important features for forecasting. Results demonstrate that when trained with the selected features, the mean absolute percentage error (MAPE) of PBNN is improved by $46.9\%$, and $73.9\%$ for Islamabad and San Diego city datasets, respectively. Furthermore, a literature comparison of the PBNN is also performed for robustness analysis. Source code and datasets are available at https://github.com/Ubaid014/Parallel-Boosting-Neural-Network/tree/main

## Introduction

The extensive use of fossil fuels is causing fast depletion of their reserves as they account for $80\%$ of total world energy consumption^1^. At the current rate of utilization, reserves of coal, oil, and natural gas are expected to be exhausted in 148, 43, and 61 years, respectively^2^. Moreover, this widespread consumption has significant environmental consequences, with fossil fuel-based power plants contributing 75% of global $CO_2$ emissions^3^. According to the study, $CO_2$ emission in India has increased by $50\%$ resulting in 1.2 million premature deaths because of the air pollution^4^. The rapidly deteriorating environment and depleting fossil fuels have brought researchers’ interest to energy transition options. These alarming statistics underscore the urgent need for sustainable energy transitions to mitigate environmental degradation and address the finite nature of fossil fuel reserves.

Renewable energy resources (RERs), particularly solar energy, represent a promising solution to these challenges. Solar energy is abundant, clean, and environmentally sustainable, making it a viable alternative to fossil fuels. Recognizing this potential, many countries have set ambitious targets to increase solar energy integration into their power systems. According to the International Renewable Energy Agency (IRENA), the global solar generation capacity is projected to reach 8500 gigawatts (GW) by 2050^5^. Pakistan aims to achieve 25% and 30% integration of variable renewable energy (VRE) into its electrical grid by 2025 and 2030, respectively^6^. Similarly, China plans to expand its photovoltaic (PV) generation capacity to 450 GW by 2030 and 1300 GW by 2050^7^. The United States targets a utility-scale PV capacity of 127 GW by 2050^8^, while Belgium aims for a 37% share of RERs in its electricity mix by 2030^9^.

Despite its vast potential, the intermittent nature of solar energy poses significant challenges to its integration into power grids. Weather variations introduce uncertainties that complicate the operation of PV-integrated systems, necessitating accurate solar irradiance (SI) forecasting. SI forecasting spans various time horizons, including ultra-short-term, very short-term, short-term, medium-term, and long-term, depending on the application^10^. However, forecasting SI remains a complex task due to its inherent non-linear dynamics influenced by weather factors such as temperature and pressure^11^. These non-linearities hinder traditional forecasting methods from accurately capturing the dynamic relationships in SI data.

Short-term SI forecasting is particularly critical for the economic and reliable operation of PV power plants. For instance, in^12,13^, statistical models, autoregressive moving average (ARMA) and integrated autoregressive moving average (ARIMA) were proposed for SI forecast. In^14^, the artificial neural network (ANN) model with feature selection technique was studied. The suitable features were extracted using a gamma test (GT) and genetic algorithm (GA). The ANN model with feature selection technique was compared with the conventional ANN model, which was trained on all parameters. An improvement of $10-15\%$ has been reported achieved by incorporating GT and GA. In^15^, a deep learning model, probabilistic solar irradiance Transformer (ProSIT) has been proposed for global horizontal irradiance forecasting for Brighton and Groningen cities. The novel approach has been compared with additive quantile regression (AQR), quantile regression forest (QRF), and probabilistic temporal convolution network (TCN) using q-risk and mean absolute scaled error (MASE). The proposed approach outperforms other models with MASE of 0.846 and 0.807 for Groningen and Brighton, respectively. Recent studies have explored advanced techniques for improving forecasting accuracy. The authors in^16^, presented a hybrid approach by integrating the gated recurrent unit (GRU) with a multivariate empirical mode decomposition technique (MEMD). The MEMD removes the non-linearities present in the data. The authors also implemented the principle component analysis (PCA) for data preprocessing. The proposed technique was tested in four different locations in India. The findings demonstrated the superior performance of MEMD-GRU over other deep-learning networks. In^17^, a deep long short-term memory with recurrent neural network (LSTM-RNN) has been put forward for 24-h interval SI prediction. Datasets of six locations: Jena, Golden, Basel, Jeju, Busan, and Incheon, were used for the models’ assessment. The LSTM-RNN was benchmarked against FFNN and the “Persistence” model using mean absolute error (MAE) and root mean square error (RMSE). Results illustrated that the LSTM-RNN draws less error than FFNN and “Persistence” models. The best results are recorded for Golden City with the MAE and RMSE of 36.90, and 60.31 $W~m^{-2}$, respectively.

Most of the literature reported for SI forecasting relies heavily on deep-learning networks, which stems the motivation of this work. The deep learning networks have reported satisfactory performances; however, due to the requirements of large datasets and high computational overhead, their applications are limited in resource-constrained environments. The hidden layers of deep learning networks help in learning the intrinsic patterns of datasets. However, with multiple hidden layers, the networks are prone to overfitting, limiting the scalability on test data. Moreover, deep learning networks have many hyperparameters like number of neurons, learning rate, the optimizer, activation function, etc. The optimized setting of these hyperparameters is also a difficult task.

For different forecasting problems, decision tree (DT) algorithms have performed competitively with deep learning networks. Tree-based learning architectures help DT algorithms outperform deep learning networks in computational time. However, these models lack scalability and generalizability. When DT models are tested on multiple datasets, they report different predictive performances. These algorithms are also sensitive to hyperparameter tuning. Varying the hyperparameter settings can lead to significant changes in forecasting performances.

The literature reports different hybrid approaches integrating data processing techniques and hyperparameter tuning methods to address the challenges with conventional methods. These approaches help in data redundancy and optimized hyperparameter configuration, improving the model’s predictive performance. Most studies used data dimensionality algorithms based on linear transformation for the SI forecasting task. These dimensionality algorithms measure the linear relationship between external and targeted variables, while neglecting the non-monotonic relation between the variables. Although these processing techniques improve the input data, the learning capability of the model is not improved. Therefore, a holistic approach is required that can measure the monotonic and non-monotonic relation among the variables, effectively learn the intrinsic SI patterns with less computational constraints, and adapt to varying conditions to ensure generalizability.

Considering these challenges, we propose a novel parallel computing-based framework consisting of DTs and neural networks. The integrated approach leverages the strengths of DTs and neural networks while enhancing the prediction accuracy, generalizability, and computational burden, making the model less dependent on the dataset size. The proposed approach also utilized an improved data dimensionality reduction algorithm that measures both linear and nonlinear relation among the variables, enhancing predictive performance of the network. The main contributions of the study are:

1. A parallel computing-based integrated approach is proposed for day-ahead SI forecasting. The proposed parallel boosting neural network (PBNN) utilizes the DT algorithms, random forest (RF), extreme gradient boosting (XgBoost), and categorical boosting (CatBoost) as the base learners, and their outputs are passed to the feedforward neural network (FFNN). The FFNN dynamically assigns the weights to the prediction of the base learners and gives the final forecast. This holistic approach effectively captures the temporal relationship in the data and provides a nuanced prediction that aligns closely with the observed data.
2. To capture the monotonic and non-monotonic relationship between the SI and external weather features, a mutual information (MI) algorithm is proposed. The algorithm’s efficacy is highlighted by training and evaluating the proposed PBNN model on all features and selected features proposed by MI.
3. The robustness analysis of the proposed model is performed in this study by implementing the model on two geographical datasets and comparing it with state-of-the-art methodologies reported in the literature.
4. The computational time comparison, including both convergence and inference times, between the proposed PBNN and models used for comparative analysis has also been reported in the study.

The remainder of the manuscript is structured as: section “Background” discusses the literature work reported for SI forecasting with the research gap, section “Methods and preliminaries” describes methods and preliminaries, models’ configurations are presented in section “Models’ configurations”, results and discussion are provided in section “Results and discussion”, section “Challenges, limitations and future work” discusses challenges, limitations and future work and section “Conclusions” outlines Conclusions.

## Background

The SI forecasting models in the literature are usually classified into two main types: physical and data-driven^18^. Numerical weather prediction (NWP), a physical method, uses differential equations for prediction tasks. The amount of available data and variations in weather conditions can affect the prediction performance of NWP. The effectiveness of NWP degrades with changing weather conditions^19^. Precise measurements of the weather parameters such as solar angle, cloud cover and aerosols are essential for physical methods. However, measuring these parameters at the required spatial and temporal resolution is challenging and resource-intensive. The data-driven models use historical SI data which may contain external weather parameters such as pressure, humidity, temperature, etc. The data-driven models are further classified into statistical, artificial intelligence, and hybrid networks. The physical methods can be integrated with data-driven approaches but this hybridization increases the complexities regarding parameters’ optimization and computational burden.

The ARMA which is a statistical model has been presented for solar power forecasting in^12^. In this study, the smart persistence (SP) has been compared with ARMA and the reported results validate the effectiveness of ARMA with 1-h interval prediction. Another variant of the AR model, ARIMA, is employed on the dataset of Abu Dhabi in^13^. The RMSE and coefficient of determination ($R^2$) are used to evaluate the model’s performance. The RMSE and $R^2$ found to be 72.88 $W~m^{-2}$ and $88.63\%$, respectively. The authors in^20^ performed a comparative analysis of the weather research and forecasting (WRF) approach with persistence, exponential smoothing (ES) and seasonal autoregressive integrated moving averages (SARIMA). Compared to other techniques, the WRF method recorded superior forecasting performance. The statistical models are computationally efficient but their drawback of not processing the data’s non-linearities degrades their forecasting performances.

Machine learning and deep learning networks provide better forecasting results because they tend to learn the non-linearities of data patterns accurately. These networks are applied in different fields like classification, computer vision, image processing and natural language processing^21^. In^22^, a comparative study of FFNN with ARIMA, MC bayesian interference, and k-Nearest Neighbour (KNN) has been studied. The dataset of Ajaccio meteorological station, France, are used for methods training. Results demonstrated the superiority of FFNN and recorded a normalized root mean square error (NRMSE) of $21\%$. In^23^, the LSTM model was proposed for SI forecasting for Florida’s PV power plant. The authors implemented the Pearson correlation coefficient (PCC) for parameters’ selection and K-mean clustering algorithm for sky-type classification. A day-ahead SI forecasting using the LSTM-RNN with a clustering approach is performed on the datasets of three locations: Jena, Basel, and Golden by the authors in^18^. The K-mean clustering technique is used to divide the days into cloudy and sunny. The performance of the LSTM-RNN model on three datasets is also compared with the FFNN, support vector machine (SVM), and Persistence approaches. Results have illustrated that LSTM performs better than other models on all three datasets. The authors in^24^ have studied multi-channel CNN, multi-head CNN and encoder-decoder LSTM networks for day-ahead SI forecasting. The model’s performances have been evaluated using RMSE, MAE, mean absolute percentage error (MAPE), and NRMSE. Results demonstrated that the encoder-decoder LSTM network recorded the best day-ahead SI forecast. The deep learning networks outperform other statistical models due to their hidden layer feature which helps in data pattern learning. However, these networks are not energy-efficient because of their computational burden. Their requirement for large-size input data also hinders their applications.

In recent years, boosting algorithms have been applied for SI forecasting. By developing weak models such that each of them addresses the weakness of the preceding, boosting algorithms seek to increase the predictive power^25^. A comparative analysis between adaptive boosting (AdaBoost) regressor, random forest (RF), and linear regression is presented by the authors in^26^ for SI forecasting with exogenous features. The dataset is collected from the HI-SEAS meteorological station and the models’ performances are evaluated using RMSE, MAE, and MSE. The results revealed that the AdaBoost performed better than other models as the recorded RMSEs for AdaBoost, RF, and linear regression are, 135.77, 164.76, and 195.4 $W~m^{-2}$, respectively. In^27^, the XgBoost model is compared with SVM for a 1 h interval forecasting. Findings have demonstrated the superiority of XgBoost over SVM. Moreover, the convergence and inference time of both models are also compared. The total computational time of 3.07 and 31.61 are recorded by the Xgboost and SVM, respectively.

To achieve better forecasting accuracy hybrid models have also been proposed in the literature in which different algorithms work in conjunction. One algorithm may be used for classification tasks and the other for forecasting. To improve the forecasting accuracy of the NWP model, the authors in^28^ integrated the NWP with a gradient-boosting algorithm. Four error measurement techniques: RMSE, MAE, mean square error (MSE), and MAPE, were used to test the prediction accuracy. The proposed technique recorded the RMSE of 6.6, 6.2, and 6.3 $W~m^{-2}$ for winter, summer, and spring seasons, respectively. The authors in^29^ have hybridized the LSTM model with the seasonal clustering forecasting technique (SCFT). The data from six geographical areas were collected from NSRDB. With SCFT, the datasets were divided into 4 types. The authors then implemented the K-mean clustering algorithm to further classify the data into cloudy, sunny, and rainy hours. The obtained clustered data was used to train the LSTM model. The RMSEs of 16.83, 13.48, 15.09, 17.05, 16.03, and 14.99 $W~m^{-2}$ were recorded for six different locations.

In^30^, the hybridization of LSTM with particle swarm optimization (PSO) was performed. The dataset consists of weather variables of Tainan city alongside other spatio-temporal features of its four neighboring cities. The PSO algorithm was used for hyper-parameter optimization of the LSTM model. Moreover, the LSTM model was compared with the multilayer neural network (MNN). Findings illustrate that the PSO-LSTM model outperforms MNN with recorded $R^2$ of 0.950, 0.946, 0.957, and 0.949 for winter, spring, summer and autumn seasons, respectively. A Transformer based multi-model framework was proposed in^31^. First, the temporal data was processed through the Informer model. Then the authors used a Vision Transformer in the subsequent stage for processing sky image data. Lastly, a cross-modality attention method with a generative decoder was used to investigate the coupling correlation and multi-step forecasting. The proposed technique has been tested on the data of Golden Colorado with an interval forecast of 10 min.

Considering the above discussion, deep learning networks are widely used for SI forecasting. These networks have reported good forecasting performance. However, problems concerning computational resources, and extensive datasets limited their applications. Moreover, with deep hidden layer structures, deep learning networks are also prone to overfitting. The DT algorithms reported higher convergence time than deep learning networks. However, they lack scalability and are sensitive to hyperparameter tuning. The existing hybrid methodologies improve SI forecasting through data dimensionality reduction and efficient hyperparameter tuning algorithms. Mostly, the data dimensionality reduction algorithms used in SI forecasting deal with the linear relationship while leaving the non-monotonic relation among the variables intact. However, in such hybrid methodologies, the forecasting model remains the same as these data preprocessing algorithms only modify the input data and do not address the shortcomings of the forecasting method. It indicates that the limitations of the forecasting model regarding continuously changing data patterns and scalability analysis persist. Therefore, an integrated approach is required that improves the forecasting model’s structure and data processing for the efficient learning of intrinsic SI patterns.

To address the challenges, we propose a parallel computing-based framework, PBNN which is composed of three DT algorithms and a neural network. In the data preprocessing, the MI algorithm is used for dimensionality reduction. The PBNN first utilizes Categorical Boosting (CatBoot), Xgboost, and RF as base learners. The input data is passed to these algorithms in a parallel way. After the training, the base learners provide forecasts, which are stacked in the intermediary forecast matrix. This matrix is then passed to FFNN where the neural network makes the dynamic weight adjustment with intermediary predictions and provides the final forecast. The performance of the PBNN is evaluated through RMSE, MAE, MSE, MAPE, NRMSE, root mean square relative error (RMSRE), mean absolute relative error (MARE), and root mean square percentage error (RMSPE). The comparative analysis of the PBNN with state-of-the-art methodologies demonstrated its scalability and robustness.

## Methods and preliminaries

**Fig. 1:**
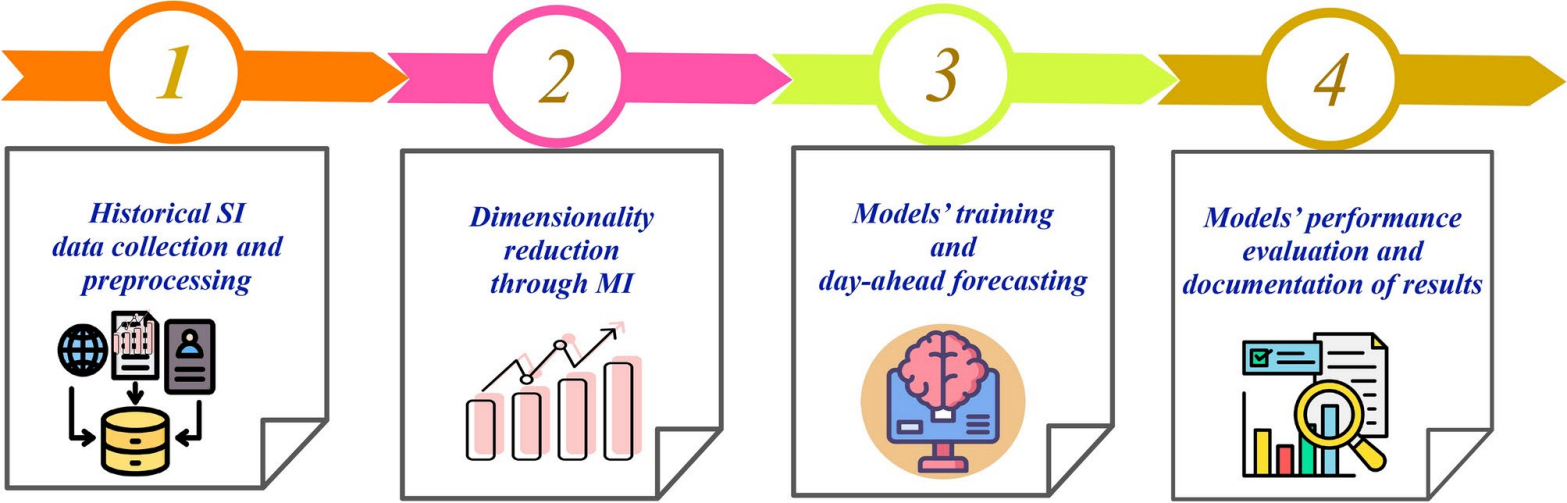
Complete workflow of the study.

In Fig. 1, the methodology of this study is demonstrated and described as follows:

1. First, historical SI data from two different locations are collected, which are enriched with external weather parameters.
2. In the data preprocessing step, data scaling and dimensionality reduction are performed. The “Standardscaler” is applied for data scaling and MI highlights the appropriate features.
3. A training dataset is used to train PBNN and benchmarked techniques.
4. Lastly, different error metrics are used to assess the models’ performance for 24-h interval forecast, and the outcomes are reported.

Further elaboration of these steps is provided in the subsequent sections.

### Extreme gradient boosting (XgBoost)

The XgBoost is a type of DT algorithm used for classification and regression problems. The main difference between XgBoost and gradient boosting algorithms is in the improvement mechanism of weak learners. The XgBoost algorithm uses multithread-mechanism and histogram-based learning to enhance the running speed and models’ performance^32^. To reduce complexity, a level-wise DT development architecture is developed. Second-order Taylor expansion defines XgBoost loss function^33^.

1 $$\begin{aligned} L(t)\approx \sum _{i=1}^{n}(L(y_i,y'_{(i-1)}+g_if_i(x_i)+ (1/2) h_if^2_i(x_i)) \end{aligned}$$

2 $$\begin{aligned} g_i= \ f'(t)=\ \frac{\partial L(y_i,y^{'(t-1)})}{\partial y^{'(t-1)}} \end{aligned}$$

3 $$\begin{aligned} h_i= \ f''(x)=\ \frac{\partial ^2 L(y_i,y^{'(t-1)})}{\partial y^{'(t-1)}} \end{aligned}$$

The prediction at $(i-1)$ instance is denoted by $y^{\prime }_{(i-1)}$.

### Categorical boosting (CatBoost)

CatBoost algorithm is also one of the variants of GBDT developed by Yandex researchers and engineers and has applications in different fields such as recommendation systems, self-driving cars, personal assistance, forecasting tasks, etc.^10^. The CatBoost model is developed for handling categorical features differentiating it from other GBDT algorithms. Oblivious trees that are created in sequence govern the workflow of CatBoost. The subsequent tree learns from its forerunner to improve the models’ performance^34^. In Catboost, first several supporting sub-models are created iteratively such that the forerunner trains on the residual of the previous model. During the training, a set of independent random permutations is generated and the leaf values of the dedicated tree are selected through the permutation set. The gradient of the prediction can be calculated as^10^:

4 $$\begin{aligned} grad_{ab}= (\partial L (r_i,j))/\partial j, j=C_{a,r}(i) \end{aligned}$$

Where the $C_{a,r}(i)$ is the prediction of the model $C_{a,r}$ at *i* instance.

### Random forest (RF) regressor

The RF regressor was proposed by Breimen and used for classification and regression problems. RF regressor works based on multiple DT and bootstrap aggregation^35^. First, *n* different points are generated that divide the training set. Each tree makes its prediction and the output is the bootstrap aggregation of all DTs^36^. For training data $D_n={(X_1,Y_1 ),\ldots ,(X_n,Y_n)}$ with $[0,1]^d\times \Re -$ valued random variable satisfying $EY^2< \infty$ distribution criteria, RF generates randomized regression trees as ${r_{n}(x,\Theta _{m},D_{n}),m\ge 1}$. An aggregated regression estimate generates the final forecast.

5 $$\begin{aligned} r_{n}^{\prime } (X,D_n )=E_{\Theta }[r_{n}(X,\Theta _{m},D_{n})] \end{aligned}$$

The symbol $\Theta _m$ represents a random variable and $E_\Theta$, which is the expectation with $\Theta _m$ and its dependents on *X* and $D_n$.

### Long short-term memory (LSTM) network

The LSTM network is one of the variants of RNN. The RNN has two major drawbacks: vanishing gradient problem and memory retrieval over an extended period^10^. The LSTM network overcomes both of these issues because of its gating architecture. The LSTM consists of input, forget and output gates. Different activation functions integrate with the gating logic to avoid the gradient vanishing^37^. The following equations define the working of the LSTM network^37^.

6 $$\begin{aligned} Forget\, gate= f\left( \tau \right) =\ \sigma [A_fx\left( \tau \right) +B_fh\left( \tau -1\right) +u_f] \end{aligned}$$

7 $$\begin{aligned} Input\, gate= i\left( \tau \right) =\sigma \left[ A_ix\left( \tau \right) +B_ih\left( \tau -1\right) +u_i\right] \end{aligned}$$

8 $$\begin{aligned} cell= c_o\ \left( \tau \right) =\varphi [A_cx\left( \tau \right) +B_ch\left( \tau -1\right) +u_c]\end{aligned}$$

9 $$\begin{aligned} Output\, gate= o\left( \tau \right) =\sigma \left[ A_ox\left( \tau \right) +B_oh\left( \tau -1\right) +u_o\right] \end{aligned}$$

10 $$\begin{aligned} c\left( \tau \right)= f\left( \tau \right) \bigodot c\left( \tau -1\right) +i\left( \tau \right) \bigodot c_o\left( \tau \right) \end{aligned}$$

11 $$\begin{aligned} h\left( \tau \right)= o\left( \tau \right) \bigodot \varphi [c\left( \tau \right) ] \end{aligned}$$

The symbols *A*, *B*, and *u* denote the network’s weight and biases, respectively. The activation functions are represented through $\sigma$ and $\varphi$. The element-wise multiplication is shown by $\bigodot$.

### Gated recurrent unit (GRU)

The GRU is also a variant of RNN with improved computational time than LSTM because of fewer gates. The GRU consists of two gates, update and rest, that retain the information over extended periods and overcome the gradient loss^37^. The GRU working is defined by the following equations.

12 $$\begin{aligned} h_t= (1-z_t\ )\ h_{t-1}\ +z_t\ h_t'\end{aligned}$$

13 $$\begin{aligned} z_t= \sigma (W_z\ x_t+U_z\ (h_{t-1}))\end{aligned}$$

14 $$\begin{aligned} h_t^\prime= \tanh (W_h\ x_t+U(r_t\bigodot h_{t-1} ))\end{aligned}$$

15 $$\begin{aligned} r_t= \sigma (W_r\ x_t+U_r\ h_{t-1}) \end{aligned}$$

Where the GRU’s metrics are shown by *W* and *U*. For the network’s output and candidate output, $h_t$ and $h_t\prime$ symbols are used. The symbols $r_t$ and $z_t$ represent reset and update gates, respectively.

### PBNN

The proposed PBNN holistically combines the multiple boosting algorithms with a neural network-based gating mechanism for enhanced day-ahead SI forecasting. The integrated architecture of PBNN leverages the strength of bootstrap and gradient boosting averaging by training the network with three base learners: RF, XgBoost, and Catboost. These algorithms are fed with the input data, work in parallel mechanisms to learn the non-linearities and patterns of the data and provide an intermediary forecast. The intermediary forecast of each model can be denoted as

16 $$\begin{aligned} y^{\prime }_{Cat}= f_{Cat}(X)\end{aligned}$$

17 $$\begin{aligned} y^{\prime }_{Xg}= f_{Xg}(X)\end{aligned}$$

18 $$\begin{aligned} y^{\prime }_{RF}= f_{RF}(X) \end{aligned}$$

Where *X* and *y* represent the external variables and targeted feature, respectively. The *f*(*x*) denotes the intermediary prediction of the base learners. The intermediary forecasts of the base learners are then stacked in the set and fed to the feedforward neural network that works as a gating mechanism to dynamically assign weights to each base model’s prediction.

$$\begin{aligned} P = \begin{bmatrix} y^{\prime }_{\text {Cat}} \\ y^{\prime }_{\text {XgB}} \\ y^{\prime }_{\text {RF}} \end{bmatrix} \in \mathbb {R}^{n \times 3} \end{aligned}$$

Where ’*P*’ denotes the matrix of stacked intermediary forecasts.

The FFNN used in the proposed PBNN architecture has two hidden layers having 64 neurons and a relu activation function. The FFNN can be denoted by $g(P,\theta )$, where $\theta$ denotes the trainable parameters regulated by the ADAM optimizer.

19 $$\begin{aligned} g(P)=softmax(W_2.ReLU(W_1.P+b_1)+b_2) \end{aligned}$$

The output layer gives three dynamic weights corresponding to three base learners’ predictions. A softmax activation function is used to normalize the contribution of each learner in the final forecast.

20 $$\begin{aligned} w_1+w_2+w_3=1, w_i\ge 0 \end{aligned}$$

The following equation defines the final forecast

21 $$\begin{aligned} y_{final}=w_1.y^{\prime }_{Cat}+w_2.y^{\prime }_{Xg}+w_3.y^{\prime }_{RF} \end{aligned}$$

**Fig. 2:**
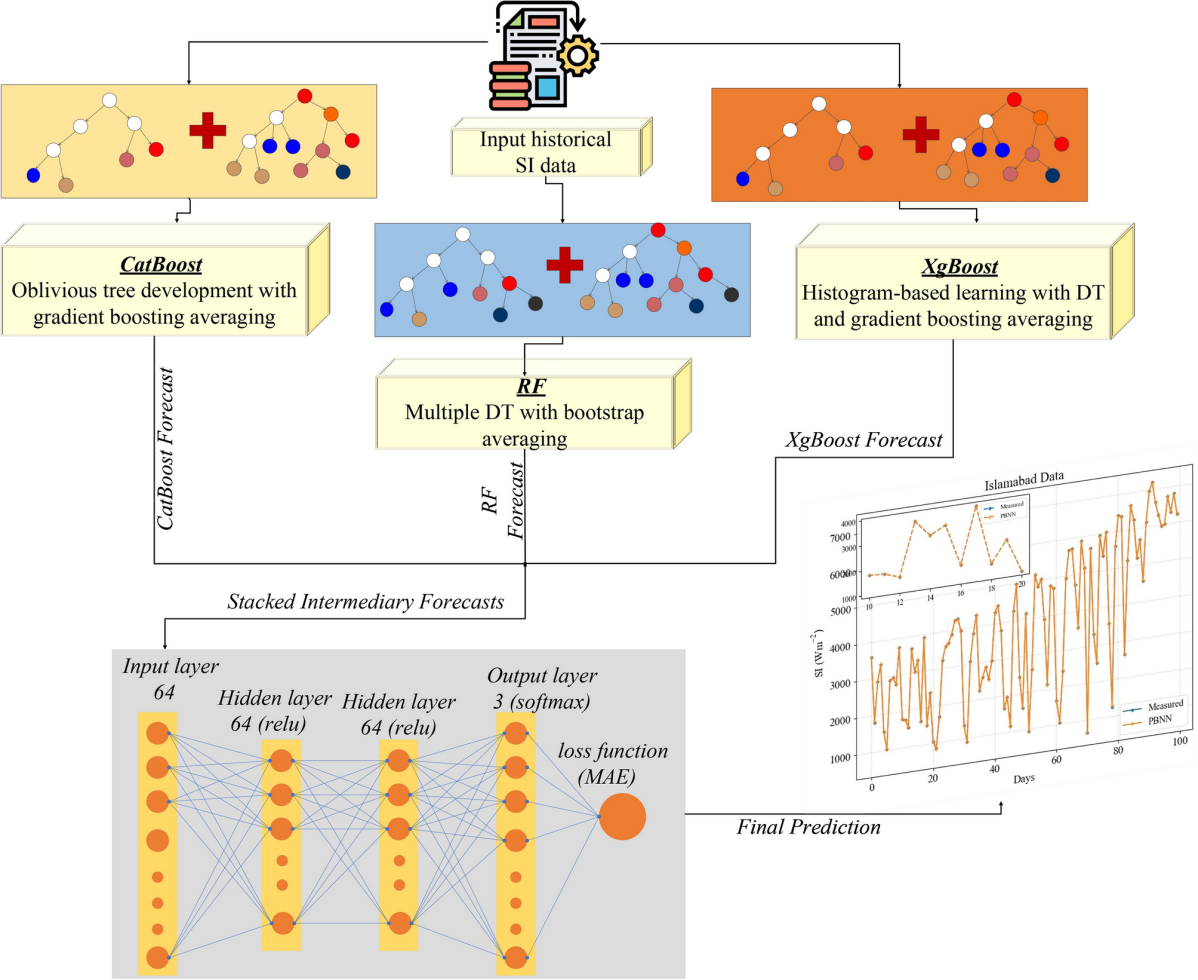
Proposed PBNN architecture.

Where $y_{final}$ represents day-ahead SI forecast. Symbols $w_1$, $w_2$, and $w_3$ denote the weights that are dynamically assigned to CatBoost, XgBoost and RF, respectively. In Fig. 2, the systematic workflow of the proposed PBNN is depicted.

## Models’ configurations

### Data overview and segmentation

This study utilizes datasets of two locations: Islamabad and San Diego. These datasets are collected from the National Aeronautics and Space Administration (NASA) database, recorded at the interval of 24 h^38^. The datasets also contain external meteorological parameters and that are: clear sky irradiance ($kW~m^{-2}$), all-sky insolation clearness index, temperature ($^\circ C$), dew point temperature ($^\circ C$), wet bulb temperature ($^\circ C$), wind direction ($^\circ$), relative humidity ($\%$), precipitation (mm/day), surface pressure (*kPa*), wind speed at 2 meters ($ms^{-1}$), wind speed at 10 meters ($ms^{-1}$), specific humidity at 2 meters (*QV*2*M*) ($g.Kg^{-1}$), all-sky surface Photosynthetically Active Radiation (PAR) ($W~m^{-2}$), clear sky surface PAR ($W~m^{-2}$) and all-sky surface Ultraviolet (UV) index. The data for Islamabad and San Diego cities ranges from 2010–2021 and 2016–2021, respectively. The $80\%$ of the data is used to train the model referred to as training dataset while the other $20\%$ of data is labeled as a testing dataset and used for performance evaluations of the models.

### Data preprocessing

Data preprocessing involves data cleaning, scaling, and feature extraction^39^. In this model, “StandardScaler” is implemented to scale the data. The “StandardScaler” normalizes the features individually by setting the mean to 0 and the variance to 1. The “StandardScaler” *S* for the variable *x* is calculated using the following equation.

22 $$\begin{aligned} S = (x-m)/ d \end{aligned}$$

Where, the symbols *m* and *d* represent the mean and standard deviation of the training sample, respectively.

#### Dimensionality reduction with MI

The selection of appropriate features is also important for the model’s performance. The proposed study has implemented the MI algorithm to select the subset of external features having more prognostic power for predicted variables.

The MI algorithm measures the information of two variables regarding each other. Unlike other correlation coefficient techniques, such as PCC, the MI is more robust as it deals with the non-monotonic relation among the variables^40^. The MI measures the linear and non-linear relationship between the variables, resulting in a more improved feature selection process. The probability density function can define the MI between two variables^41^.

23 $$\begin{aligned} M(I;O) = \iint {N_{I,O}(i,o) \log \left( \frac{N_{I,O}(i,o)}{N_{I}(i) P_{O}(o)} \right) \, di \, do} \end{aligned}$$

Entropy for the random variable I can be described as

24 $$\begin{aligned} E(I) = -\int {N_{I}(i) \log N_{I}(i) \, di} \end{aligned}$$

The MI can be defined as

25 $$\begin{aligned} M(I,O) = E(I) - E(I|O) = E(O) - E(O|I) \end{aligned}$$

The uncertainty of a random variable can be represented through entropy. Equation (25) describes the MI criterion of maximizing the *M*(*I*, *O*) for feature selection by searching the feature subset that reduces the uncertainty regarding the targeted variable *O*.

### Hyperparameter tuning

The optimized parameter tuning of a model plays a key role in improving its forecasting accuracy, computational speed and memory requirements. Different algorithms are introduced in the literature to tune hyperparameters. This study employs the randomized search (RS) algorithm for parameter tuning. The RS is an efficient algorithm to explore a large combination of hyperparameter settings. It randomly samples the hyperparameters from a defined search space over a number of trails, in the present case 10, making it less exhaustive and computational than the grid search algorithm, which explores all the possible values of the search space. Table 1, reports the hyperparameters selection process.

**Table 1:** Hyperparameter optimization with selected search space.

Models	Hyperparameter	Search space	Setting for Islamabad dataset	Setting for San Diego dataset
XgBoost	Estimators	{100, 200, 300}	300	300
	Learning rate	{0.01, 0.03, 0.1 ,0.5}	0.5	0.1
	Colsample bytree	{0.6, 0.8, 0.9, 1}	0.9	1
	Max. depth	{6, 9, 12}	12	9
	Subsamples	{0.7, 0.8, 0.9, 1}	1	0.7
	Tree method	{“gpu hist,” “approx”}	“approx”	“approx”
CatBoost	Estimators	{1000, 1250, 1500}	1250	1500
	Maximum depth	{2, 4, 6, 8}	2	2
	Learning rate	{0.1, 0.3, 0.01, 0.001}	0.3	0.1
	L2 leaf regularization	{0.2, 0.5, 1, 3}	0.5	0.1
RF	No. of estimators	{20, 60, 100, 120}	60	60
	Min. sample split	{2, 5}	5	2
	Min. sample leaf	{1, 2}	1	2
	Max. samples	{0.5, 0.75}	0.5	0.5
	Max. features	{0.2, 0.6, 1.0}	8	8
	Max. depth	{2, 6, 8}	8	6
LSTM	No. of units	{32, 64, 128, 256}	256	128
	Optimizers	{“Adam,” “RMSprop,” “SDG”}	RMSprop	Adam
	Learning rate	{0.01, 0.001, 0.0001}	0.01	0.0001
	Hidden layers	{1, 2, 3}	2	3
	Dropout	{0.1, 0.2, 0.3, 0.4} 0.1		0.3

### Performance metrics

This study makes use of five error measurement techniques: RMSE ($W~m^{-2}$), MSE ($(W~m^{-2})^2$), MAE ($W~m^{-2}$), MAPE ($\%$) and NRMSE ($\%$), to evaluate the performance of predictive models^37^.

26 $$\begin{aligned} RMSE= \sqrt{\frac{1}{N}\sum _{I=1}^{N}(X_I-Y_I)^2} \end{aligned}$$

27 $$\begin{aligned} NRMSE= \frac{RMSE}{mean(Y_I)}*100 \end{aligned}$$

28 $$\begin{aligned} MAE= \frac{1}{N}\sum _{I=1}^{N}{|X_I-Y_I|} \end{aligned}$$

29 $$\begin{aligned} MSE= \frac{1}{N}\ \sum _{I=1}^{N}{((X_I-Y_I)}^2 \end{aligned}$$

30 $$\begin{aligned} MAPE= \frac{1}{N}\sum _{I=1}^{N}\frac{|X_I-Y_I|}{X_I}*100 \end{aligned}$$

31 $$\begin{aligned} \text {RMSRE}= \sqrt{\frac{1}{N} \sum _{i=1}^{N} \left( \frac{X_I- Y_I}{X_I} \right) ^2} \end{aligned}$$

32 $$\begin{aligned} \text {MARE}= \frac{1}{N} \sum _{i=1}^{N} \left| \frac{X_I - Y_I}{X_I} \right| \end{aligned}$$

33 $$\begin{aligned} \text {RMSPE}= \sqrt{\frac{1}{N} \sum _{i=1}^{N} \left( \frac{X_I - Y_I}{X_I} \times 100 \right) ^2} \end{aligned}$$

Where, $X_I$ and $Y-I$ represent observed and forecasted values, respectively.

**Fig. 3:**
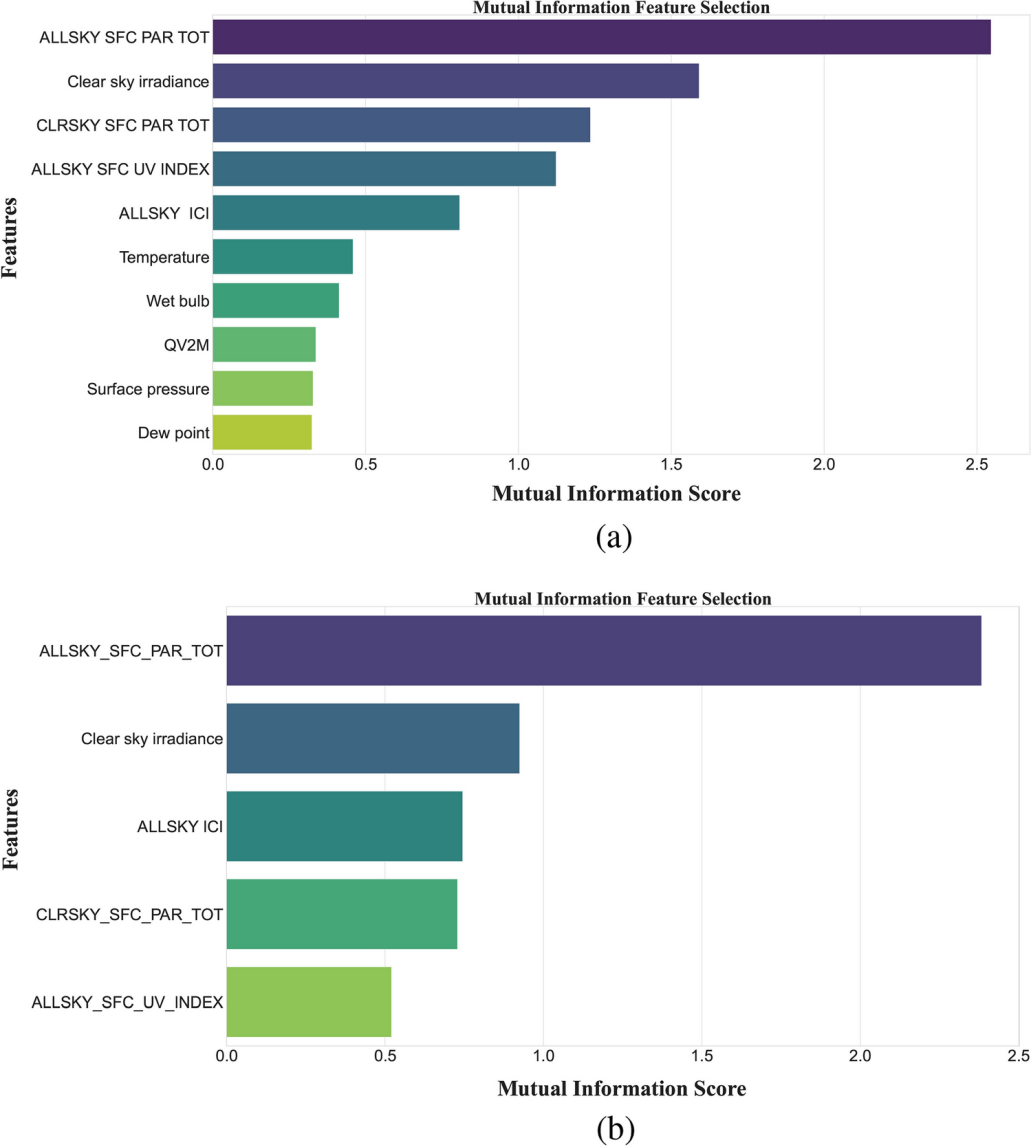
Feature selection by MI (**a**) for Islamabad dataset, (**b**) for San Diego dataset.

## Results and discussion

The simulations of the PBNN approach and benchmarked techniques are implemented on a MacBook Apple M1 chip with 8GB RAM using the “Python” 3.10.9 version and results are documented in this section.

### Feature selection importance

In addition to the SI, the datasets of each location also contain 14 meteorological parameters. The MI algorithm is implemented to extract the most appropriate features’ subset. In the proposed study, we have set the threshold of MI score to 0.2. The selected suitable features for Islamabad city dataset are: all-sky surface PAR ($W~m^{-2}$), clear sky irradiance ($KW~m^{-2}$), clear sky surface PAR ($W~m^{-2}$), all-sky surface UV, all-sky insolation clearness index, temperature ($^\circ C$), wet bulb temperature ($^\circ C$), QV2M ($g.Kg^{-1}$), surface pressure (*KPa*), and dew point ($^\circ C$).

The features, all-sky surface PAR ($W~m^{-2}$), clear sky irradiance ($KW~m^{-2}$), all-sky insolation clearness index, clear sky surface PAR ($W~m^{-2}$) and all-sky surface UV are highlighted as appropriate features for SI forecasting by MI algorithm for San Diego city dataset. Figure 3 depicts the feature selection results for each dataset.

**Fig. 4:**
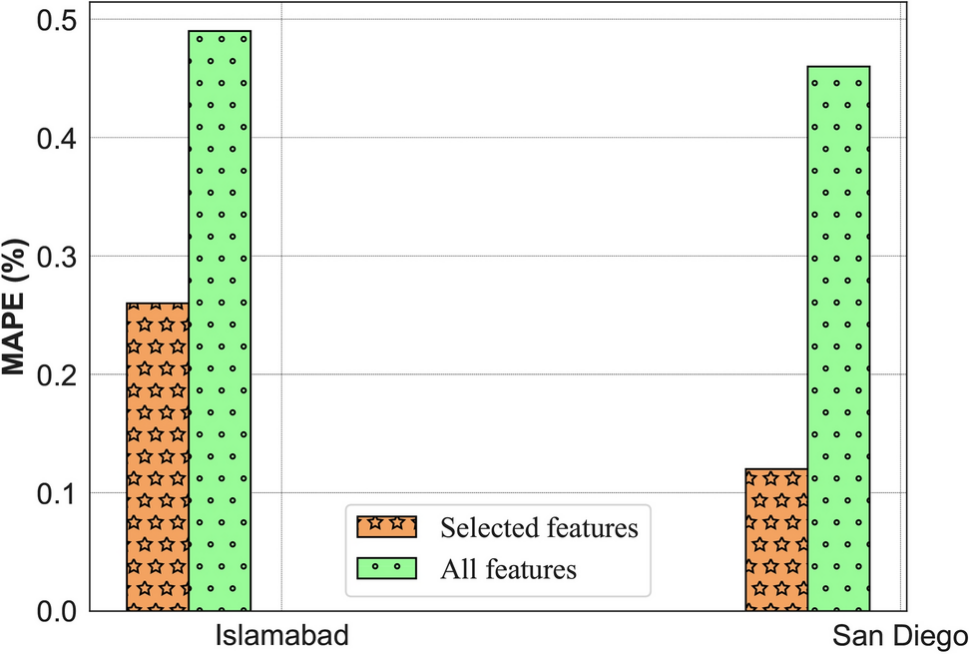
Illustration of feature selection importance with the MAPE bar plot.

To highlight the importance of dimensionality reduction for data-driven models, we have evaluated the performance of the proposed PBNN against a selected feature subset and all features. The comparative results are presented in Table 2. Results demonstrate that the MAPE of PBNN improves by $46.9\%$ and $73.9\%$ when trained with a feature subset spotted by the MI algorithm. In Fig. 4, the improvement in the MAPE of the PBNN by the MI algorithm is illustrated through a bar plot.

**Table 2:** Improvement in the predictive performance of PBNN with MI-based feature selection.

Location	Models	RMSE	MAE	MSE	MAPE	NRMSE
		($$W~m^{-2}$$)	($$W~m^{-2}$$)	($$W~m^{-2})^2$$	(%)	(%)
Islamabad	Selected features	14.06	8.36	197.77	0.26	0.29
	All features	25.18	17.39	633.97	0.49	0.52
San Diego	Selected features	17.23	5.26	296.84	0.12	0.32
	All features	36.74	21.86	1349.92	0.46	0.7

### Models’ comparison

Table 3 presents the performance evaluation of PBNN and other models selected for the comparative analysis. All the techniques are trained with the selected features to ensure a fair comparison among the models.

**Table 3:** Findings of the comparative analysis between the PBNN and other techniques.

Location	Model	RMSE	MAE	MSE	MAPE	NRMSE	RMSRE	MARE	RMSPE
		($(W~m^{-2})$)	($(W~m^{-2})$)	($(W~m^{-2})^2$)	($(\%)$)	($(\%)$)			($(\%)$)
Islamabad	CatBoost	30.67	21.47	940.69	0.68	0.64	0.014	0.0068	1.41
	XgBoost	22.98	13.17	528.37	0.54	0.48	0.015	0.0054	1.55
	RF	16.13	9.61	260.13	0.31	0.34	0.008	0.003	0.81
	LSTM	38.57	28.59	1487.91	1.04	0.79	0.025	0.01	2.54
	GRU	55.21	47.04	3048.05	1.42	1.15	0.022	0.014	2.18
	**PBNN**	**14.06**	**8.36**	** 197.77**	**0.26**	**0.29**	0.006	0.002	0.56
San Diego	CatBoost	38.68	29.69	1496.52	0.63	0.74	0.008	0.006	0.86
	XgBoost	19.19	10.51	368.22	0.23	0.37	0.004	0.002	0.48
	RF	26.26	11	689.45	0.24	0.5	0.006	0.002	0.65
	LSTM	75.38	47.41	5682.16	0.99	1.45	0.015	0.009	1.56
	GRU	78.98	50.78	6239.16	1.08	1.51	0.017	0.011	1.73
	**PBNN**	**17.23**	**5.26**	**296.84**	**0.12**	**0.32**	0.004	0.001	0.48

**Fig. 5:**
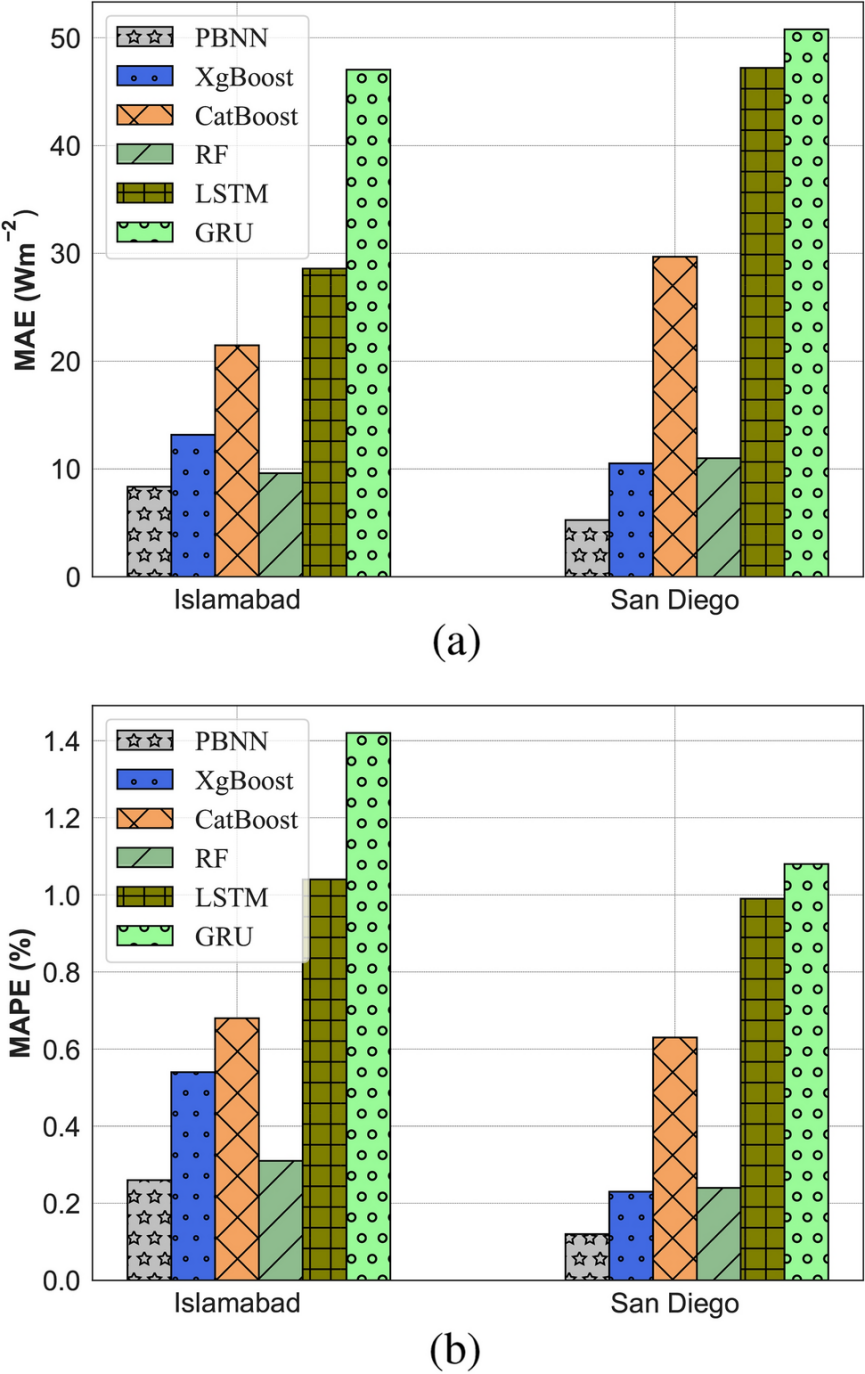
Error evaluation representation through bar plot. (**a**) MAE bar plot, (**b**) MAPE bar plot.

Findings demonstrate that the proposed PBNN approach gives better forecasting results on each dataset than other models. The RMSE, MAE, MSE, MAPE, and NRMSE of 14.06, 8.36, 197.77, 0.26, and 0.29, respectively are recorded by PBNN for the Islamabad city dataset. The RMSE, MAE, MSE, MAPE, and NRMSE of the proposed integrated approach are 17.23, 5.26, 296.84, 0.12, and 0.32, respectively for San Diego City data. In Fig. 5, the MAE and MAPE of the models are depicted through a bar plot.

The results also demonstrate the vulnerability of the deep learning networks in case of fewer data points by recording a weak performance on the San Diego city dataset compared to another dataset. Unlike deep learning networks, the proposed PBNN consistently demonstrates robust forecasting performance across both datasets, making it less dependent on individual data points.

**Fig. 6:**
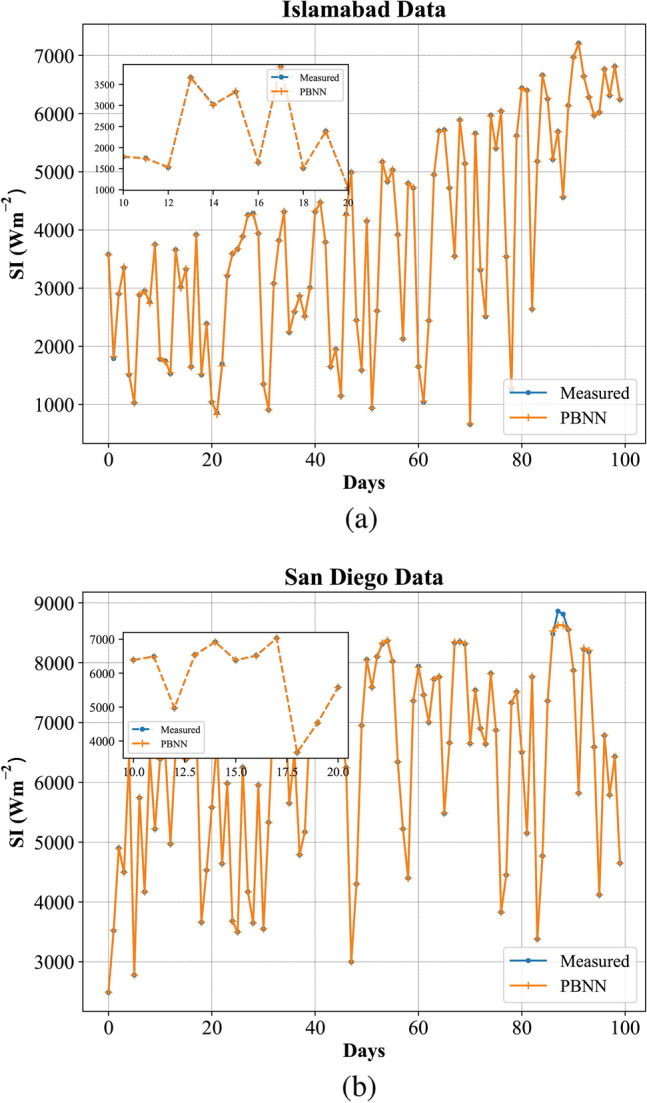
Demonstration of accurate curve fitting by the proposed PBNN (**a**) Islamabad, (**b**) San Diego.

**Fig. 7:**
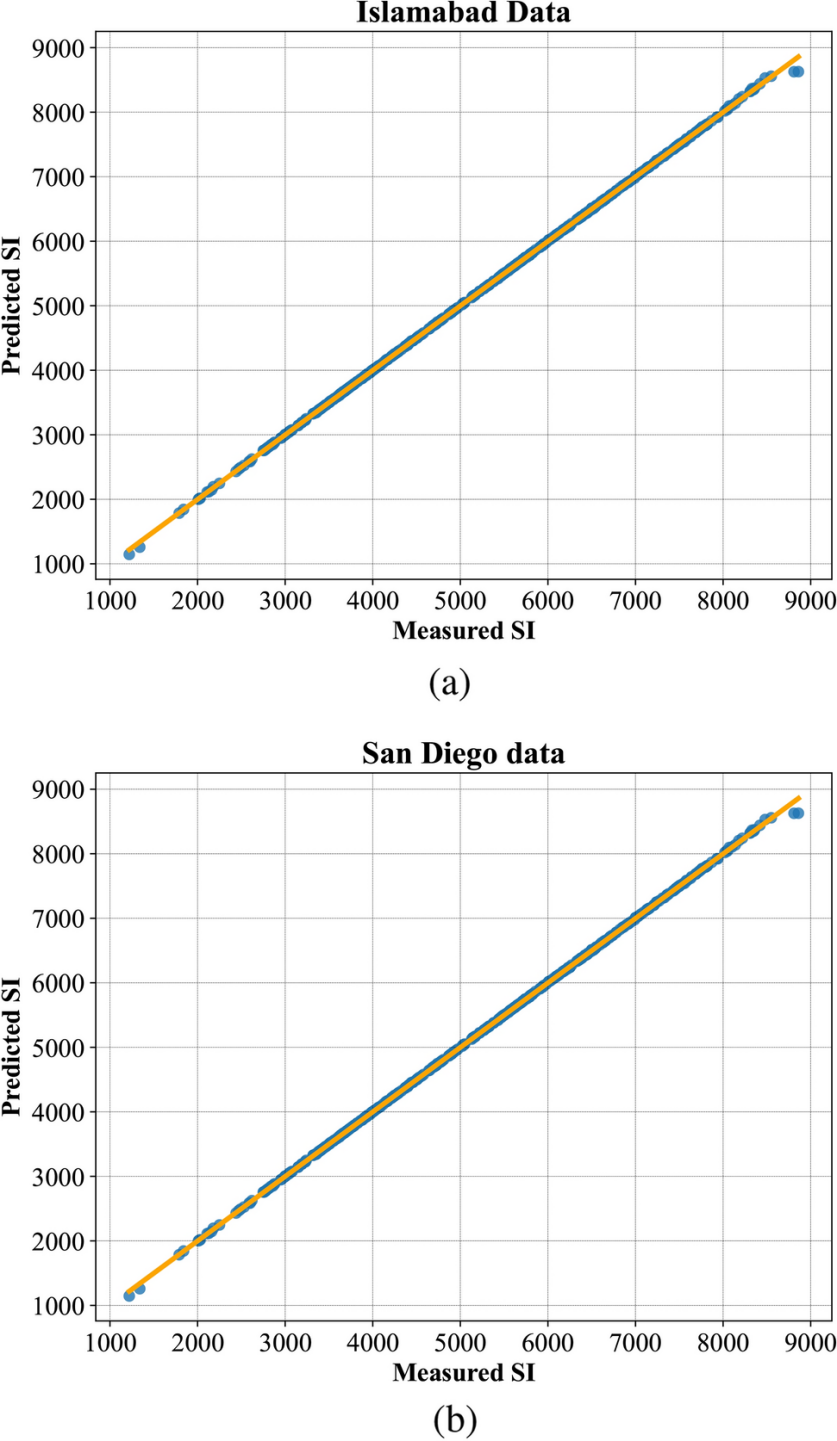
Regression line plot of proposed PBNN (**a**) Islamabad, (**b**) San Diego.

In Fig. 6, the PBNN predicted SI curves are compared with observed values. The graphical representation illustrates that the proposed technique quite fits the measured curves. This shows the effectiveness of the PBNN for day-ahead SI forecasting. Figure 7 depicts the fitted line plots for measured and predicted SI by PBNN. In a line plot, the model’s predictive performance is evaluated by the fitness of points over the regression line. Thus, Fig. 7, illustrates the superiority of PBNN as the predicted outputs lie close to the regression line.

The computational time comparison between the models is reported in Table 4. The findings illustrate that the individual boosting algorithms have better computational time due to their tree-based structure. The deep learning networks reported the worst computational time among the models. The proposed PBNN approach integrates three boosting algorithms with FFNN, which results in higher computational time than individual boosting algorithms. However, the PBNN has less computational time than GRU and LSTM networks. The trade-off in higher computational time of PBNN than individual boosting algorithms is justified by improved forecasting outcomes of the proposed approach.

**Table 4:** Computational time comparison between the models.

Location	Models	Convergence time	Inference time	Computational time
		(s)	(s)	(s)
Islamabad	CatBoost	0.459	0.033	0.492
	XgBoost	0.247	0.003	0.25
	RF	0.285	0.009	0.294
	LSTM	284.637	0.466	285.103
	GRU	243.045	0.471	243.516
	**PBNN**	**9.906**	**0.084**	**9.99**
San Diego	CatBoost	0.308	0.001	0.309
	XgBoost	0.381	0.002	0.383
	RF	0.144	0.002	0.146
	LSTM	56.238	0.292	56.53
	GRU	50.159	0.229	50.388
	**PBNN**	**3.342**	**0.036**	**3.379**

In Table 5, the performance of PBNN and the state-of-the-art models are reported when trained with all features. Findings of Table 5 highlight the superior performance of PBNN to other models. However, with the dimensionality reduction through MI, the forecasting outcomes are further improved.

**Table 5:** Forecasting performances of the networks trained with all features.

Location	Model	RMSE	MAE	MSE	MAPE	NRMSE
Islamabad	CatBoost	30.2	22.19	912.3	0.72	0.63
	XgBoost	52.69	37.42	2776.4	1.13	0.49
	RF	26.53	18.5	703.86	0.56	0.55
	LSTM	93.44	16.99	8731.81	3.95	1.94
	GRU	80.99	53.74	6560.94	1.96	1.68
	**PBNN**	**25.18**	**17.39**	**633.96**	**0.49**	**0.52**
San Diego	CatBoost	43.98	31.68	1934.79	0.69	0.84
	XgBoost	49.31	34.48	2432.18	0.69	0.94
	RF	31.71	53.86	2900.91	0.64	1.03
	LSTM	21977.2	148.25	115.41	2.39	2.88
	GRU	113.81	65.18	1.34	1.08	2.18
	PBNN	36.74	21.86	1349.92	0.46	0.7

### Threshold setting

**Fig. 8:**
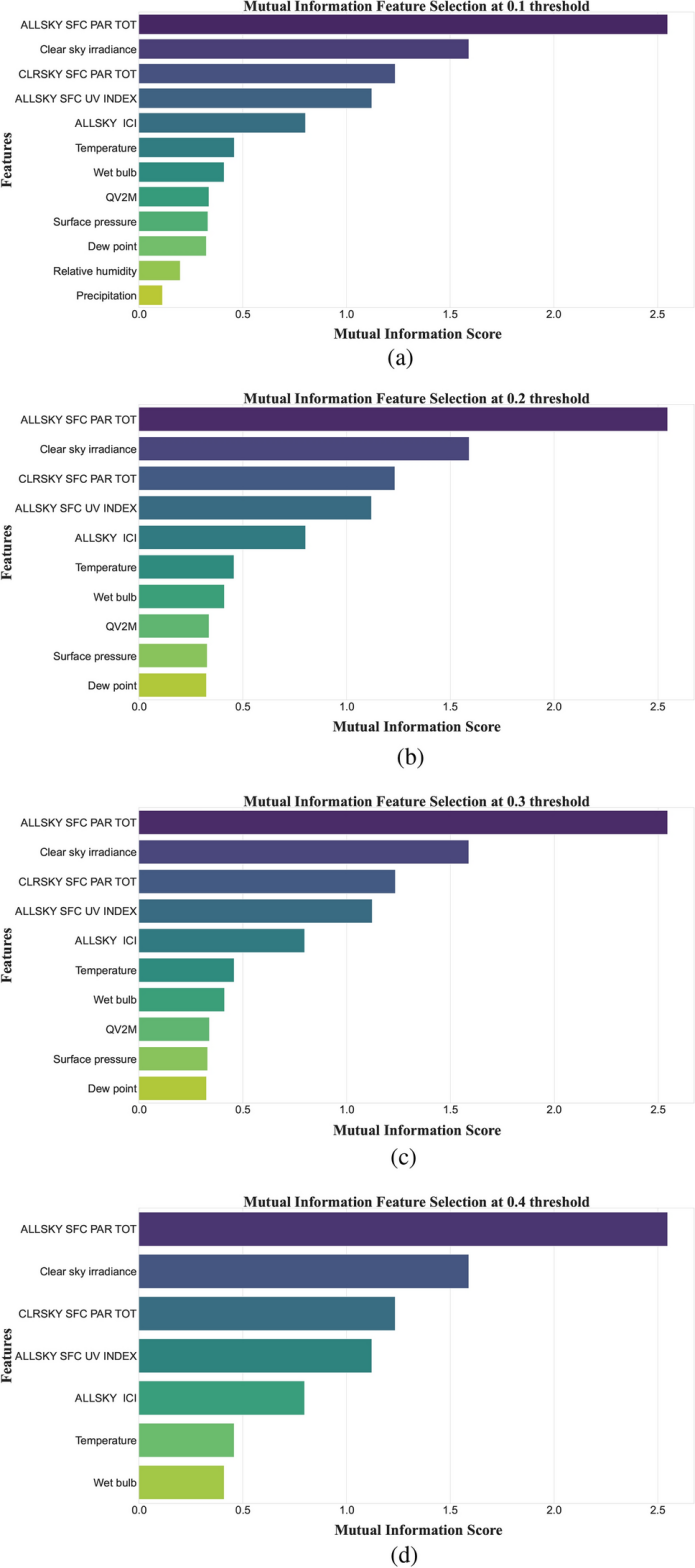
Feature selection by MI at different threshold levels.

The threshold level for MI is selected iteratively. First, the MI algorithm is applied with a threshold of 0.1. The key external features identified by MI are retained, and the performance of the PBNN is evaluated. Then, the threshold level is gradually increased to 0.2, 0.3, and 0.4. The PBNN’s performance is assessed at each threshold by training the model with the features indicated by each respective threshold. Figure 8 illustrates the MI output at different thresholds.

The Fig. 8 depicts that at threshold 0.2 and 0.3, the MI selected the same external variables. Furthermore, at threshold 0.1, 0.2, and 0.4 the MI filtered 12, 10, and 7 external features, respectively, as the most appropriate for the model’s learning. The model trained with a variable from the threshold level of 0.4 reported the better computational time as it has fewer features to interpret than other threshold levels. In Table 6, the performance of the PBNN at threshold 0.1, 0.2 and 0.4 is reported.

The findings indicate that the best results are reported at 0.2 threshold. In case of 0.1 threshold, the MI filtration of external variable is insufficient resulting in lower forecasting outcome. At 0.4 threshold, the PBNN filters key variables that negatively impact the PBNN’s performance. The most optimal performance is achieved at 0.2 threshold.

**Table 6:** PBNN forecasting performance at different thresholds.

Location	Threshold level	RMSE	MAE	MSE	MAPE	NRMSE	Computational time
**Islamabad**		($W/m^2$)	($W/m^2$)	$(W/m^2)^2$	(%)	(%)	(s)
	**0.1**	19.2	12.49	368.76	0.36	0.39	10.02
	**0.2**	14.06	8.36	197.77	0.26	0.29	9.986
	**0.4**	19.67	11.14	386.93	0.45	0.41	9.84

### Literature comparison

In this study, we have compared the performance of the PBNN with other approaches presented in the literature for SI forecasting. In^17^, a hybrid model of LSTM with RNN for six different locations has been put forward for SI prediction. The best forecasting result is achieved for the Golden City dataset. In^18^, authors enhanced the LSTM-RNN forecasting capability through weather classification. In^29^ and^16^, the authors have proposed hybrid methodologies of deep learning networks with clustering and decomposition strategies for hour-ahead SI forecasting. In this work, for the literature comparison, we trained the PBNN on the datasets used in^16–18,29^, and reported the results in Table 7. Findings validate the robustness of the proposed PBNN approach as it records superior performance than the techniques already reported in the literature.

**Table 7:** Comparison of proposed hybrid approach with other models presented in the literature.

Ref	Journal publisher	Location	Model	Interval	RMSE	MAE
					($W~m^{-2}$)	($W~m^{-2}$)
^17^	Energies	Golden, USA	LSTM-RNN	24hr	60.31	36.9
18	Applied Sciences	Golden, USA	K-mean-LSTM-RNN	24hr	43.41	17.6
**Proposed study**		**Golden, USA**	**PBNN**	**24hr**	**4.76**	**3.35**
29	IEEE-TII	Tripoli, Libya	SCFT-LSTM	1hr	13.48	10.05
**Proposed study**		Tripoli, Libya	**PBNN**	**1hr**	**3.92**	**0.80**
16	Renewable Energy	JodhPur, India	MEMD-GRU	1hr	31.92	22.99
16	Renewable Energy	New Delhi, India	MEMD-GRU	1hr	36.25	25.65
**Proposed study**		**JodhPur, India**	**PBNN**	**1hr**	**11.06**	**4.12**
**Proposed study**		**New Delhi, India**	**PBNN**	**1hr**	**15.55**	**4.74**

**Fig. 9:**
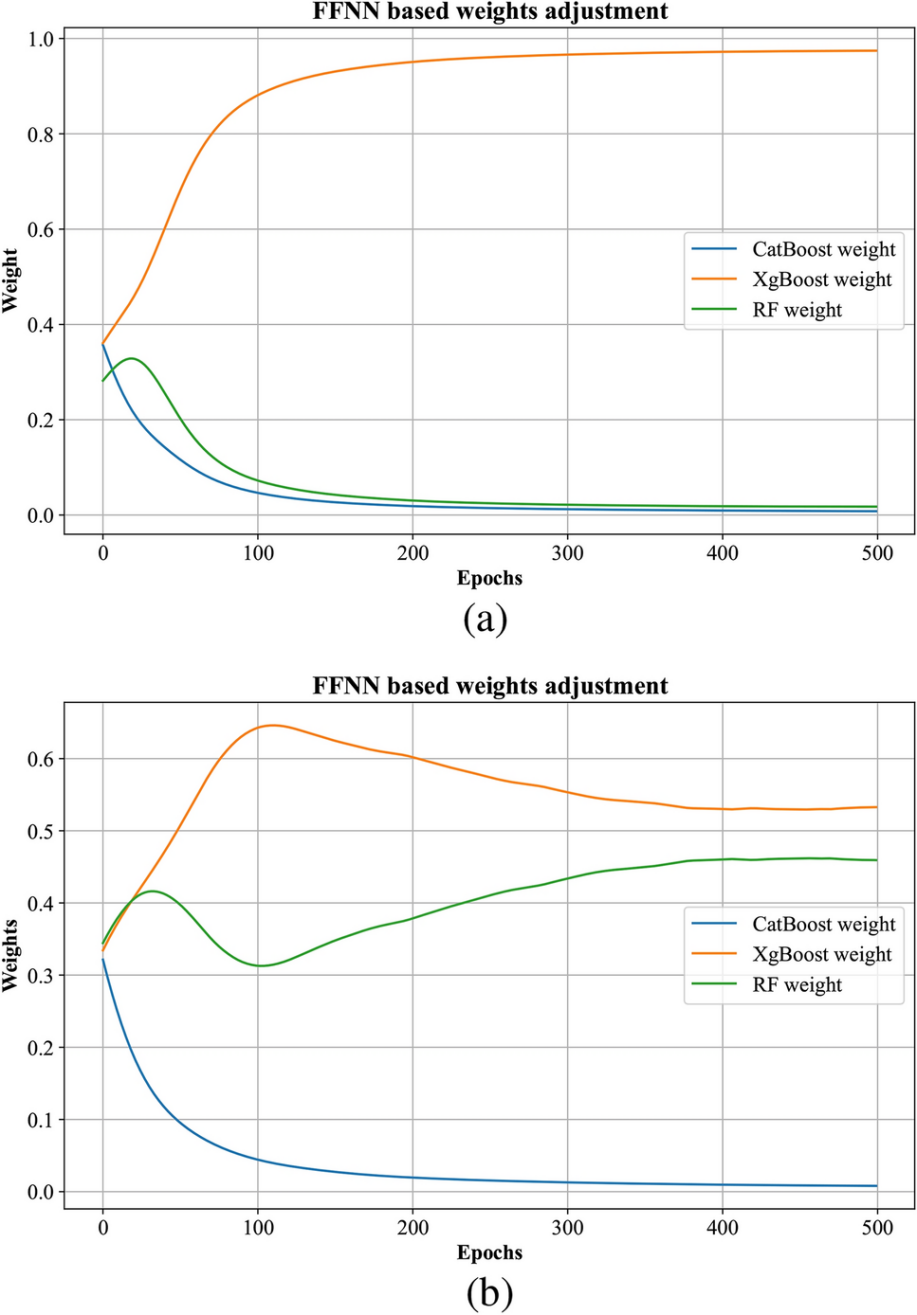
Dynamic assignment of weights to base learners’ prediction with FFNN (**a**) For Islamabad city data, (**b**) For San Diego city data.

The proposed PBNN records superior forecasting performance due to its holistic architecture, which integrates different techniques for more accurate SI prediction by addressing the challenges of intermittent nature. The PBNN first employs three boosting algorithms and computes the non-linearities of the data points by bootstrap and gradient-boosting averaging. The CatBoost uses sequential DT with ordered boosting for data learning. The XgBoost implements regularization parameters with histogram learning to provide its intermediary forecast, while the RF averages its multiple DTs’ output to layer 2. The FFNN dynamically assigns weights to each base learner prediction which overcomes boosting algorithms’ generalizability and scalability issues. Most of the stacking techniques are based on fixed averaging. For instance, in mean averaging, the predictions of the base models are stacked using simple averaging. These averaging techniques give equal importance to the base learner models regardless of how they interpret the data. When the model, which fails to learn the intrinsic data patterns, is given equal importance as the model that learns data patterns effectively, the overall performance of the meta learner degrades. In the proposed study, the FFNN assigns relative importance to different base learners’ predictions using the softmax function. The softmax function normalized the weight sum to one, representing the relative importance of base learners at the given forecasting period. If one boosting algorithm is assigned higher weights, it indicates that at this particular interval, this model is capturing the data effectively. Moreover, in Fig. 9, the different weights assigned by FFNN to the boosting algorithm over the epochs are represented. The parallel computing of the boosting algorithms at the base learner level also reduces the computational overhead of the network. The PBNN effectively learns the hidden data patterns and provides more accurate SI predictions that align closely with the observed data by integrating multiple strategies.

The proposed PBNN has reported more accurate and scalable forecasting performance on different geographical datasets compared to state-of-the-art deep learning networks and standalone gradient-boosting algorithms. Moreover, the comparative analysis performed with recently reported techniques in the literature further validates the PBNN’s robustness in learning the dynamic and intermittent data pattern of SI. The PBNN is trained and evaluated on the same open public dataset used in other reported studies for direct performance comparison under identical data conditions and therefore, providing a reliable benchmarking for assessing its effectiveness for SI forecasting.

## Challenges, limitations and future work

The interpretability of weight adjustment based on FFNN is the critical challenge of the proposed study. The FFNN dynamically assigns weights to the base learners’ prediction. However, due to the black box nature of the neural networks, understanding of weight assignment at specific intervals is difficult. Another challenge lies in the overfitting of FFNN when dealing with noisy data. If the input data contain outliers which are not scaled properly, the FFNN assigns misleading weights, degrading the network’s performance. Improving the practicability of the proposed PBNN requires addressing these challenges.

Despite reporting superior performance by the proposed PBNN, this study has certain limitations. Due to limited computational resources, the models are tuned with small search spaces. This approach helps to achieve the model’s satisfactory performance within the computational feasibility. However, within the small search space, the most optimal configuration of hyperparameters may not be discovered. Optimizing the key parameters of models with large search space potentially improves their learning capabilities. Several techniques, such as reinforcement learning, Bayesian optimization, and Optuna, can be implemented to discover the optimal setting of hyperparameters over a large search space^42^. Another limitation of the study is that the PBNN has not been assessed under severe weather conditions, such as storms and cloud cover. These extreme weather conditions bring drastic changes in the SI patterns. The conventional methods often fail to adapt to the rapid fluctuations and uncertainties in irradiance level introduced by extreme weather conditions.

Future work will focus on evaluating the model’s performance on island datasets. The islands have extreme weather events and testing the model on these datasets further validates its scalability and robustness. Moreover, the focus will be on enhancing the network’s performance by incorporating more advanced neural networks for dynamic weight adjustment, and other ensemble learning techniques, such as light gradient boosting machine (LGBM) and AdaBoost. Additionally, the network’s performance will be tested on real-world problems, for instance, unit commitment and economic dispatch.

## Conclusions

The intermittent nature of SI requires an accurate forecasting method for the reliable operation of PV-integrated systems. Day-ahead forecasting of SI deals with efficient management of energy reserves. Therefore, in this study, we have presented a novel parallel computing with a dynamic weight adjustment technique for day-ahead SI forecasting. The proposed strategy is called PBNN and consists of three boosting algorithms that are trained in parallel, and their intermediary forecasts are stacked and passed to the FFNN. The neural network then assigns different weights to the intermediary predictions of the base learners for the final output forecast. The study has also presented an MI-based feature selection technique to improve the data-driven model’s predictive abilities. The MI measures the monotonic and non-monotonic relation among the variables and provides the subset of the features with more forecasting power concerning SI. The MAPE of PBNN improves by $46.9\%$ and $73.9\%$ when trained with the feature subset passed by the MI algorithm. The proposed PBNN has been trained on two geographical datasets for scalability analysis and benchmarked with state-of-the-art techniques. Findings illustrate that the PBNN outperforms the models used for comparative analysis. The RMSE, MAE, MSE, MAPE, and NRMSE of 14.06, 8.36, 197.77, 0.26, and 0.29 are recorded, respectively by PBNN for the Islamabad city dataset. In the case of San Diego, the RMSE, MAE, MSE, MAPE, and NRMSE of the proposed PBNN model are found 17.23, 5.26, 296.84 0.12, and 0.32, respectively. Furthermore, the study has highlighted the robustness of the PBNN by comparing it with the techniques already reported in the literature.

## Supplementary Information

Supplementary Information.

## Data Availability

The datasets used and/or analyzed during the current study are available from the first author on reasonable request (Ubaid Ahmed: ubaidahmedrj334@gmail.com).
