# Supplementary figures and images for: Parallel boosting neural network with mutual information for day-ahead solar irradiance forecasting
Source: Sci Rep. 2025 Apr 4;15:11642. doi: 10.1038/s41598-025-95891-1 (PMC11971244; doi:10.1038/s41598-025-95891-1)

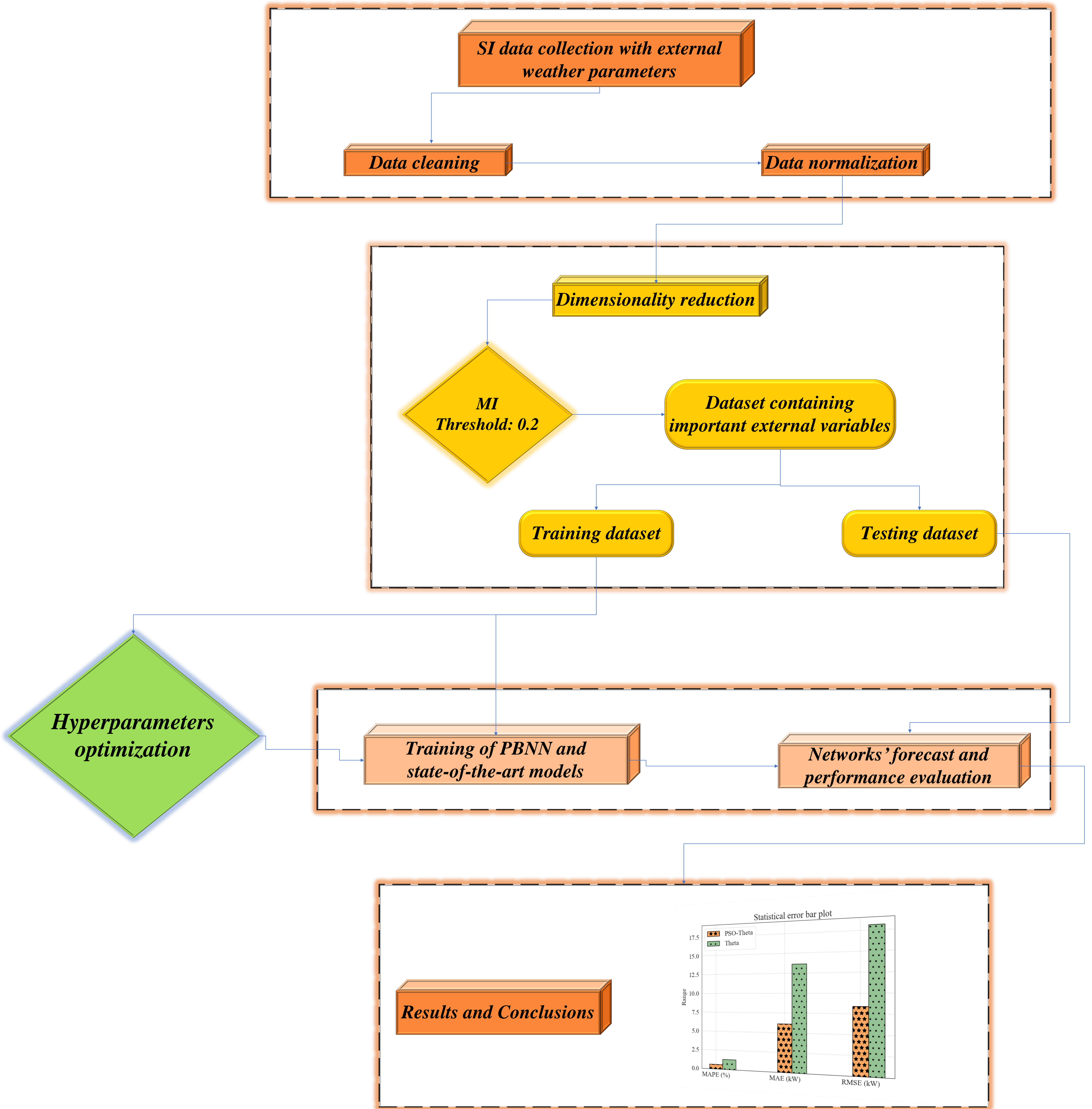

Supplement: Supplementary file 1 — Supplementary Information. [file 41598_2025_95891_MOESM1_ESM.pdf]
